# Personals

**Published:** 1913-01

**Authors:** 


### PERSONALS.

---

Our readers are requested to send notes for this and similar news departments.

---

Drs. Jacob E. Helwig and T. C. P. Barnard, Health Officers of Pendleton and North Tonawanda, respectively, attended the annual conference of Health Officers of the state, at Syracuse, Dec. 5-7.

---

Dr. C. V. Wilson of Tonawanda, has been ill with pneumonia.

---

Dr. E. H. Mudge of Westfield, was in Buffalo early in December.

---

Dr. Howard L. Prince has become associated with Dr. Ralph R. Fitch, in the practice of orthopaedic surgery. Office at 365 East Ave., Rochester.

---

Dr. C. Haase has moved his office to the south-east corner of Lake and Water Sts., Elmira. Dr. F. Haase, the dentist, is associated with him.

---

Dr. Herman G. Matzinger of Buffalo was appointed medical council to Hickey, the confessed pervert and murderer of a little boy at Lackawanna. Dr. B. Ross Nairn served as alienist to Justice Brown, Rrs. James W. Putnam and Nelson W. Wilson as alienists for the prosecution.

---

Dr. James Wright Putnam of Buffalo, had his automoblie stolen early in December. It was found the next morning with the head lights smashed.

Dr. J. F. Macpherson of Payne Ave., N. Tonawanda, has left for California, on account of his health, and may decide to locate permanently in one of the Pacific coast cities. Dr. Henry Lapp of Clarence has purchased Dr. Macpherson's residence and will assume his practice.

---

Dr. Earl G. Danser of Buffalo, Post Mortem Examiner, while returning in his automobile from investigating a death at Akron, steered into a shed to avoid wrecking a buggy. He sustained a fracture of the wrist. Dr. Bernard H. Brady who was with him, was thrown out of the car but was not seriously injured.

---

Dr. Allen W. Holmes is now living in Akron, N. Y.

---

Dr. Carl Alsberg, a chemist in the Bureau of Drugs and Plants, has been appointed by President Taft, on the approval of Sec. Wilson, to Dr. Harvey W. Wiley's position as Chief of the Bureau of Chemistry of the Department of Agriculture.

---

Miss Dorothy Hastings, a graduate of the Boston City Hospital, is in charge of the Nurses' Home of the Buffalo Homoeopathic Hospital.

---

Dr. J. H. Glass of Utica, has started on a tour around the world, expecting to return about May 1, 1913.

---

Dr. R. B. Lyons of Elmira, who was operated upon in November, for appendicitis, has resumed practice.

---

Drs. Henry and Mabel Flood of Elmira, have moved their offices to the Realty Building.

---

Dr. A. L. Benedict wishes to state that his views on diet are neither so interesting nor so original as certain press notices of lectures in Buffalo and Rochester, might indicate. This is not intended as a slur upon the reporters but it is obviously impossible in a short article, prepared from extemporaneous notes, and for the purpose of a newspaper, to present the qualifications and explanations necessary as a basis of criticism by those familiar with the subject from the medical standpoint.

---

Dr. George W. Kosmak of 23 E. 93d St., N. Y. City, is collecting notes on pregnancy occurring after oophorectomy. The attention of our readers is asked.

Drs. O. H. Babbitt and Wm. E. Walsh of Auburn, have been re-elected jail and coroner's physician, respectively.

---

Dr. J. H. Whitbeck of Cayuga and Dr. A. D. Stewart of Port Byron, have been elected coroner's physicians for Cayuga County.

---

Dr. Jack Killen of Binghamton, sustained serious injuries by colliding with a telegraph pole, in avoiding a pedestarian.

---

Dr. J. A. Lichty of Pittsburg, called on the editor, Dec. 13.

---

Dr. John H. Grant has moved from Albany to 202 W. 79th St., New York City.

---

Dr. Clifford A. Orr has been elected Treasurer of the Park Club.

---

Dr. Hyatt Regester of Buffalo, has moved to 615 Elmwood Ave.

---

Dr. Bernard Cohen of Buffalo, spent three weeks in Atlantic City after attending the Clinical Surgical Congress in New York.

---

Dr. Herbert M. Tolfree, U. S. N., was the guest of Dr. H. G. Hopkins of Buffalo during the holidays.

---

Dr. Charles H. Frazier of Philadelphia, was in Rochester on Dec. 7th, the guest of Dr. W. A. Keegan.

---

Dr. and Mrs. J. M. Ingersoll of Rochester have been away for some weeks in the Bermuda Islands.

---

Dr. L. A. Whitney of Rochester, has just bought a home on Alexander Street.

---

Dr. E. L. Hanes of Rochester, recently purchased a home in East Main Street.

---

Dr. H. A. Sodden of Rochester, is occupying his home recently completed at the corner of Dewey Ave. and Albemarle St.

---

Dr. E. H. Wolcott has just returned from a short trip to New York.

Dr. E. W. Mulligan has returned from a trip in the South.

---

Dr. Mary Innis Denton, formerly of Buffalo, now of Western College, Oxford, O., spent the holidays in Buffalo.

---

Drs. W. W. Britt and R. P. Reagan of North Tonawanda, spent the holidays with relatives out of town.

